# Assessment of unconventional antimicrobial compounds for the control of ‘*Candidatus* Liberibacter asiaticus’, the causative agent of citrus greening disease

**DOI:** 10.1038/s41598-020-62246-x

**Published:** 2020-03-25

**Authors:** Christopher L. Gardner, Danilo R. da Silva, Fernando A. Pagliai, Lei Pan, Kaylie A. Padgett-Pagliai, Ryan A. Blaustein, Marcelo L. Merli, Dan Zhang, Cécile Pereira, Max Teplitski, Jose X. Chaparro, Svetlana Y. Folimonova, Ana Conesa, Salvador Gezan, Graciela L. Lorca, Claudio F. Gonzalez

**Affiliations:** 1 Microbiology and Cell Science Department, Genetics Institute, Institute of Food and Agricultural Science, University of Florida, Gainesville, Florida United States of America; 2 Soil and Water Sciences Department, Genetics Institute, Institute of Food and Agricultural Science, University of Florida, Gainesville, Florida United States of America; 3 Fruit Tree Breeding and Genetics, Horticultural Sciences Department, Institute of Food and Agricultural Science, University of Florida, Gainesville, Florida United States of America; 4 Plant Pathology Department, Institute of Food and Agricultural Science, University of Florida, Gainesville, FL 32611 USA; 5 School of Forest Resources and Conservation, Institute of Food and Agricultural Science, University of Florida, Gainesville, Florida United States of America

**Keywords:** Plant sciences, Microbiology

## Abstract

In this study, newly identified small molecules were examined for efficacy against ‘*Candidatus* Liberibacter asiaticus’ in commercial groves of sweet orange (*Citrus sinensis*) and white grapefruit (*Citrus paradisi*) trees. We used benzbromarone and/or tolfenamic acid delivered by trunk injection. We evaluated safety and efficacy parameters by performing RNAseq of the citrus host responses, 16S rRNA gene sequencing to characterize citrus-associated microbial communities during treatment, and qRT-PCR as an indirect determination of ‘*Ca*. L. asiaticus’ viability. Analyses of the *C. sinensis* transcriptome indicated that each treatment consistently induced genes associated with normal metabolism and growth, without compromising tree viability or negatively affecting the indigenous citrus-associated microbiota. It was found that treatment-associated reduction in ‘*Ca*. L. asiaticus’ was positively correlated with the proliferation of several core taxa related with citrus health. No symptoms of phytotoxicity were observed in any of the treated trees. Trials were also performed in commercial groves to examine the effect of each treatment on fruit productivity, juice quality and efficacy against ‘*Ca*. L. asiaticus’. Increased fruit production (15%) was observed in *C. paradisi* following twelve months of treatment with benzbromarone and tolfenamic acid. These results were positively correlated with decreased ‘*Ca*. L. asiaticus’ transcriptional activity in root samples.

## Introduction

‘*Candidatus* Liberibacter asiaticus’ (CLas) is a fastidious, phloem-limited bacterium and the main causative agent of Huanglongbing (HLB), also known as citrus greening disease^1^. Since its emergence in China nearly 100 years ago, a multitude of management strategies have been implemented in an attempt to mitigate the extensive economic damage caused by this pathogen. In areas such as California, where the disease is not yet widespread, management strategies are focused on increased testing for HLB and removal of infected trees, however, the disease remains prevalent in the majority of citrus producing regions throughout the world^1–5^. Recent treatment strategies have included a combination of insecticides, antimicrobials and thermotherapy, but an effective long-term treatment, that is specific for CLas, remains elusive. In December 2018, the U.S. Environmental Protection Agency approved the use of oxytetracycline on citrus^6^; however, CDC and FDA officials have expressed concerns regarding the use of medically important broad-spectrum antibiotics in agriculture. While the use of broad spectrum antibiotics has been shown to be successful in some groves infected with HLB, phytotoxicity is also well documented in the literature, when oxytetracycline is applied at the concentrations needed for efficacy against CLas^7,8^. Taken together, the identification of novel therapeutic targets in CLas is an essential step towards the development of a safe and sustainable treatment for HLB.

We previously conducted molecular screening assays to identify small molecules that bind with high affinity to CLas proteins of interest, as a means to identify potential therapeutics for citrus greening disease. While mechanisms of pathogenicity have been well documented for numerous plant pathogens, the CLas genome does not encode genes commonly associated with pathogenicity, and virulence factors specific to CLas remain largely unknown^9–11^. Thus, we performed an in-depth analysis of the differentially expressed genes in CLas, *in planta* and in the psyllid vector, to identify therapeutic targets, where our analyses also revealed an exceptionally low number of genes encoding transcription factors in the CLas genome. The genes encoding those proteins were cloned, purified, characterized and successfully screened against the Prestwick chemical library (Prestwick Chemical, France) to identify ligands with high binding affinity. Using this methodology, we successfully identified small molecules that inhibit the activity of specific transcription factors^12–16^. Based on these previously published data, we performed field trials using benzbromarone and tolfenamic acid, the small molecules that were found to inhibit activity of the regulatory proteins LdtR and PrbP.

LdtR is a member of the Multiple Antibiotic Resistance Regulator (MarR) family of transcriptional regulators that control expression of nearly 180 genes in CLas, through interactions with regulatory sequences in promoter regions^13,15^. Analysis of the CLas transcriptome revealed *ldtR* expression was five times higher in samples collected from infected trees, when compared to samples obtained from the infected psyllid vector, suggesting an important role during plant infection and/or persistence in the citrus host^11,13^. Thermal screening assays against a small molecule library identified benzbromarone as a high affinity ligand for LdtR. The biological impact of LdtR inactivation (through interactions with benzbromarone) was subsequently validated in *Sinorhizobium meliloti* and *Liberibacter crescens*, where concerted changes in gene expression and cell physiology were observed following growth in presence of benzbromarone^13,15^. These results were consistent with morphological changes and reduced tolerance to osmotic stress observed in *ldtR* mutants^13^. Consequently, an *in vitro* model was designed to test the efficacy of benzbromarone against CLas in infected *C. sinensis* leaves, where expression of CLas *16S rRNA* and *rplJ* (50 S ribosomal subunit protein L10) decreased significantly (90.9 ± 6.1 and 97.6 ± 1.5 percent decrease, respectively; p < 0.005) following incubation with benzbromarone^13^. A decreased level of expression was also observed for *ldtR, CLIBASIA_02905* (putative LuxR family transcriptional regulator)*, CLIBASIA_04655* (chemotaxis sensory transducer)*, CLIBASIA_01505* (putative ferredoxin protein) and a hypothetical protein *CLIBASIA_01670* (35%, 24%, 51%, 24% and 47%, respectively; p < 0.05) when compared to the untreated controls^15^.

PrbP is a transcriptional accessory protein belonging to the CarD_CdnL_TRCF family, which regulates gene expression through interactions with RNA polymerase^14,17–22^. Homologs of PrbP in *Myxococcus* and *Mycobacterium* are associated with antibiotic resistance, pathogenicity, persistence, oxidative stress response and vegetative growth under starvation conditions^17,19,23–26^. Using a similar approach to LdtR, purified PrbP was screened against a chemical library, where tolfenamic acid was identified as a high affinity ligand^14^. Interactions between PrbP and tolfenamic acid were further examined *in vitro*, where tolfenamic acid was found to disrupt interactions between PrbP and DNA^12,14^. The use of tolfenamic acid as a potential antimicrobial against CLas was consequently examined in a greenhouse study using graft-inoculated citrus trees, where decreased titers of CLas (indicated by decreased gene expression for *gyrA, rplJ, prbP, rplK* and *16S rRNA*), were observed in trees that received tolfenamic acid treatments via spray applications^14^.

In summary, the search for a novel therapeutic treatment for citrus greening has, so far, relied heavily on modern molecular techniques with a limited number of new chemicals being tested in commercial groves. In this work, we report the results obtained from an exhaustive evaluation of two chemicals that were previously found to be effective against CLas using *in vitro* models as well as in a greenhouse setting^13,14^. We performed a comprehensive study to evaluate the efficacy of these compounds against HLB in large-scale field trials, and determined their impact on the microbiome, plant responsiveness and the quality and quantity of fruit produced.

## Results

### Evaluation of benzbromarone and tolfenamic acid efficacy in small citrus trees

**Figure 1:**
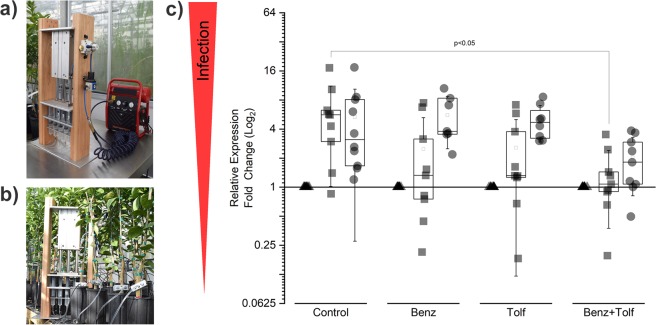
Analysis of ‘*Candidatus* Liberibacter asiaticus’ gene expression following trunk injections with benzbromarone and tolfenamic acid. (**a**) A pneumatic trunk injection system was specifically designed to deliver trunk injections with accurate pressure control to minimize tissue damage. (**b**) Injectors connected to small citrus trees. (**c**) The expression of CLas 16S rRNA in leaf tissue, was measured by qRT-PCR in trees injected with buffer only (Control), benzbromarone (Benz), tolfenamic acid (Tolf), or benzbromarone and tolfenamic acid (Benz + Tolf). Leaf tissue samples were collected for analysis immediately before injections (▲), three months post-injection (■), and six months post-injection (●). ANOVA and Tukey’s post-hoc test was performed for each time point; only relevant statistical differences are shown. All expression data was normalized to the citrus *COX* gene.

Graft-inoculated two-year-old *C. sinensis* trees were used to assess the efficacy of a single trunk injection of Benz, Tolf, and Benz+Tolf against CLas infection. Treatments were administered using a pneumatic injection system designed and built in our laboratory for this application (Fig. 1a,b). Foliar tissue samples were collected at three-month intervals for analysis by quantitative reverse transcription-polymerase chain reaction (qRT-PCR), where the mRNA levels of the CLas 16S rRNA gene were used as an indirect measurement of CLas viability and/or proliferation, as previously described^13,14^.

The combined treatment (Benz + Tolf) was effective in decreasing (p < 0.05) CLas infection when delivered by trunk injection. Lower transcription of the CLas 16S rRNA gene was observed after three months in trees that received Benz+Tolf injections (250 µM each), when compared to controls that received buffer only (Fig. 1c). While the individual Benz and Tolf injections were found to reduce CLas transcriptional activity three months post-treatment, a single injection was unable to completely eliminate the bacteria from graft inoculated trees, as indicated by elevated CLas transcriptional activity at six months post-treatment (Fig. 1c). Due to the small trunk size of the two-year-old trees used in this experiment, repeat trunk injections were not feasible.

### Trunk injections with benzbromarone and tolfenamic acid reduce CLas gene expression in mature citrus trees (small-scale field trial)

A small-scale field trial was conducted with mature citrus trees at the University of Florida Gainesville campus. The goal of this assay was to optimize the delivery method, investigate environmental impacts of each chemical, and evaluate effectiveness against CLas. The double treatment (Benz + Tolf) was not included in this assay.

**Figure 2:**
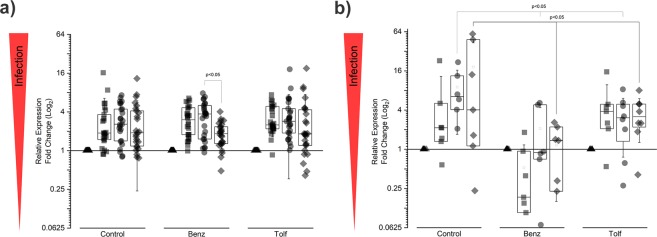
Analysis of the efficacy of Benz and Tolf treatment on ‘*Candidatus* Liberibacter asiaticus’ gene expression in mature citrus trees (small-scale field trial). The viability of CLas was analyzed in (**a**) foliar and (**b**) root tissue following trunk injections in mature (>20 year-old) citrus trees. CLas viability was indirectly inferred from mRNA levels of the 16S rRNA gene, normalized to mRNA levels of the citrus *COX* gene. Samples were analyzed before treatment (▲), and at 2 months (■), 6 months (●) and 12 months (◆) post-treatment. ANOVA and Tukey’s post-hoc test was performed for each time point; only relevant statistical differences are shown.

This assay was conducted over a period of twelve months with field-grown *C. sinensis* trees (>20 years-old). Each tree was injected at the beginning of the assay, and repeat injections were administered at 2, 6 and 12 months after the first injection. Prior to each injection, tissue samples (root and foliar) were collected for analysis by mRNA-based qRT-PCR, to determine the effect of each treatment on microbial titers. The statistical analyses of CLas gene expression in foliar tissue indicated a significant decrease (p < 0.05) for CLas 16S rRNA following four injections with Benz (Fig. 2a). Both Tolf and control groups maintained a constant level of infection during one year of treatment (Fig. 2a). The analysis of root tissue showed stronger significance values for both treatments, suggesting trunk injections favored treatment of the roots (Fig. 2b). After six months both Benz and Tolf treatments show a decrease in infection of the roots compared to the control trees (p < 0.05).

### Benz and Tolf treatments do not disrupt citrus-associated microbiota diversity

The impact of each chemical formulation, on citrus-associated microbial communities, was examined for 18 months in foliar and root tissue, via 16S rRNA gene sequencing. 115 leaf samples and 119 root samples yielded microbial communities with more than 1,000 16S rRNA gene sequences assigned to 16S rRNA of bacteria/archaea. From these 234 samples, the 3,929,605 assigned reads, with an average of 15,598 ± 584 sequences per leaf sample and 32,891 ± 1,092 sequences per root sample (mean ± SE), were included in further analyses.

**Figure 3:**
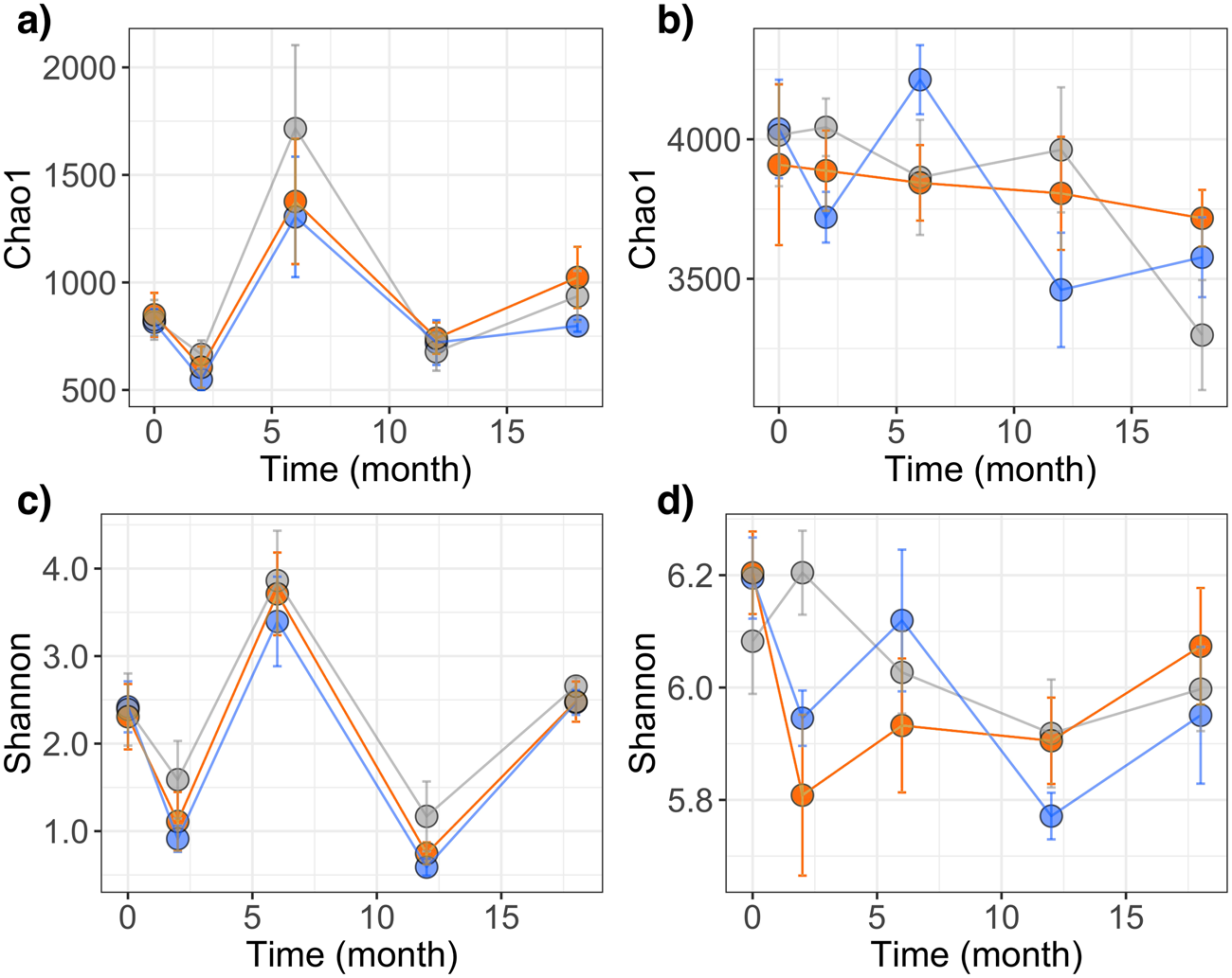
Alpha diversity fluctuations of microbiota were not induced by the treatments. (**a**) Chao1 index for leaf microbiota; (**b**) Chao1 index for root microbiota; (**c**) Shannon measure for leaf microbiota; (**d**) Shannon measure for root microbiota. Control (gray), Benz (blue), and Tolf (orange).

There were 6,987 total Operational Taxonomic Units (OTUs) that comprised the leaf microbiota; 501 ± 31 OTUs were detected in each leaf sample (mean ± SE). There were 12,162 total OTUs that comprised the root microbiota; 2,577 ± 39 OTUs were detected in each root sample (mean ± SE). Sampling time was substantially associated with the structure of leaf microbiota (ANOSIM R statistic = 0.415, significance based on 999 permutations = 0.001), though not with that of root microbiota (ANOSIM R statistic = 0.105, significance based on 999 permutations = 0.001) (Supplementary Fig. S1). Treatments did not have any detectable effects on the overall structure of the microbiota (leaves: ANOSIM R statistic = −0.013, significance based on 999 permutations = 0.972; roots: ANOSIM R statistic = 0.022, significance based on 999 permutations = 0.016) (Supplementary Fig. S1). Accordingly, the Chao1 indices significantly differed at different time points (leaf p < 0.001; root p = 0.010), but not based on treatment (leaf p = 0.505; root p = 0.940) (Fig. 3). The same trends were evident for the Shannon measure of alpha diversity, in which there were also significant differences based on time (leaf p < 0.001; root p = 0.006), but not treatment (leaf p = 0.165; root p = 0.535). Interestingly, after 18-month post treatment, there were trends for higher diversity in root microbiota of treated trees compared to that of controls (Fig. 3). Moreover, the relative abundance of *Alphaproteobacteria* in leaves and roots did not significantly differ based on time (leaf p = 0.720; root p = 0.362) or treatment (leaf p = 0.307; root p = 0.626). Within *Alphaproteobacteria*, there were 89 and 98 genera detected throughout the study, in leaf and root samples, respectively. Those with relative abundance that significantly differed based on treatment are listed in Supplementary Table S1. Thus, while treatments did not grossly disrupt alpha diversity, or adversely impact cumulative *Alphaproteobacteria* abundance, there were a few cases in which treatments may have been detrimental to other genera within the phytopathogen’s taxonomic class (Supplementary Table S1).

### Treatments suppress *Liberibacter* in root-associated microbial communities

**Figure 4:**
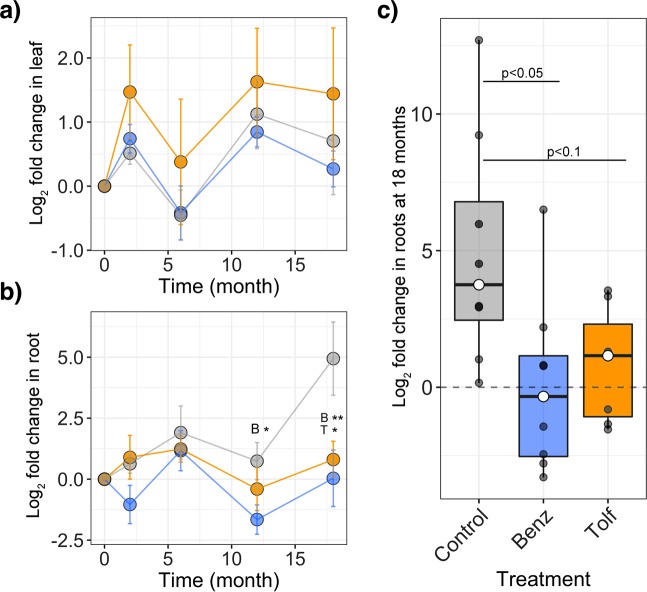
Relative levels of *Liberibacter* are affected by Benz and Tolf treatments. The log2 fold changes in the relative abundance of *Liberibacter*, relative to time zero, in trees that received Benz (blue), Tolf (orange), or the buffer Control (gray). (**a**) Leaf microbiota at all sampling points. (**b**) Root microbiota at all sampling points. ANOVA and Tukey’s post-hoc test was performed for each time point; asterisks directly above data points denote statistical significance (*p < 0.1 and **p < 0.05) for Benz or Tolf treatments (indicated by “”B” or “T”, respectively) compared to the Control. (**c**) Root microbiota at the 18-month time point. Points that fall below the dotted line correspond to trees that had a net reduction in *Liberibacter* in root communities.

At time 0, the relative abundance of *Liberibacter* among leaf microbiota was 54.7 ± 5.5% (mean ± SE), which was on average, the most abundant out of the 491 genera detected in leaf samples. In root-associated communities, the relative abundance of *Liberibacter* was 0.07 ± 0.01% (mean ± SE), which was, on average, the 152nd most abundant out of the 760 genera detected in root samples. For leaf communities, the log2 fold change in the relative abundance of the pathogen significantly differed over time (p = 0.048), but not in response to treatment (p = 0.116) (Fig. 4a). For root communities, the changes in relative abundance of *Liberibacter* differed over time (p = 0.005), with major proliferation of *Liberibacter* in control trees; treatments had suppressive effects on the log2 fold change in pathogen abundance (p = 0.001) (Fig. 4b).

At 18 months, treatments had a significant impact on log2 fold change in the relative abundance of *Liberibacter* within root microbiota (p = 0.019) (Fig. 4c). The pathogen was substantially suppressed in trees that had received Benz (Tukey adjusted p = 0.023) or Tolf (Tukey adjusted p = 0.069), compared to the control group. Interestingly, about half of the root microbiota in treated trees (n = 7 out of 16 trees) had experienced a net reduction in the relative abundance of the pathogen, while all control trees contained levels at or above their starting values (Fig. 4c).

**Figure 5:**
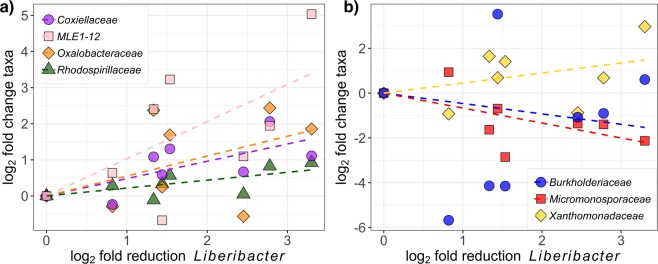
Root microbiota response to chemically-induced removal of *Liberibacter* in root-associated communities. Regression of log2 fold changes in relative abundance of *Liberibacter* compared to those of each bacterial family, after 18 months of treatment. (**a**) Bacterial families that experienced significant increases (p < 0.05) in relative abundance in response to the reduction of *Liberibacter*: (**b**) Response of the three bacterial families that were previously found to be negatively associated with *Liberibacter* in leaf microbiota.

To test the hypothesis that chemically induced removal of *Liberibacter* was associated with increased content for other key taxa (Fig. 4c), the log2 fold changes in relative abundance of *Liberibacter* were compared to those of each bacterial family with regression analyses (Fig. 5). The families that underwent significant increases in log2 fold change in relative abundance (p < 0.05) in response to the *Liberibacter* reduction, and had >0.1% relative abundance in root microbiota throughout the study (i.e., they were continuously present) were *Coxiellaceae*, MLE1-12, *Oxalobacteriaceae* and *Rhodospirillaceae* (Fig. 5a). Previously, negative associations (p < 0.05) were identified between *Liberibacter* and *Burkholderiaceae*, *Micromonosporaceae* and *Xanthomonadaceae* within leaf microbiota^27^. Here, it was tested whether these potential interactions held true in the root microbiota of trees that experienced a reduction in *Liberibacter* abundance. Of these three families, only the log2 fold change in relative abundance of *Xanthomonadaceae* appeared to increase in response to pathogen removal, however, the increase did not reach statistical significance (p = 0.095) (Fig. 5b). There were no associations between log2 fold change in relative abundance of *Liberibacter* and *Burkholderiaceae* (p = 0.488), and associations with *Micromonosporaceae* were, unexpectedly, positively correlated (p = 0.011) (Fig. 5b).

### Evaluation of the impact of each antimicrobial on the host transcriptome

Assessment of the plant response to each treatment was evaluated by RNA-seq. This assay was performed on citrus leaf samples collected immediately before the first treatment (T0) and after three months of treatment (one week after the second injection of the Benz or Tolf; T2). Tophat2^28^ was used to align the reads to the *Citrus clementina* reference genome (ncbi_gca_000493195_1_citrus_clementina_v1_0), the *C. sinensis* chloroplast sequence, and the *C. clementina* mitochondrial genome. The *C. clementina* genome was selected as a reference for read mapping instead of *C. sinensis* because of the higher quality in assembly of the *C. clementina* genome compared to *C. sinensis*.

**Figure 6:**
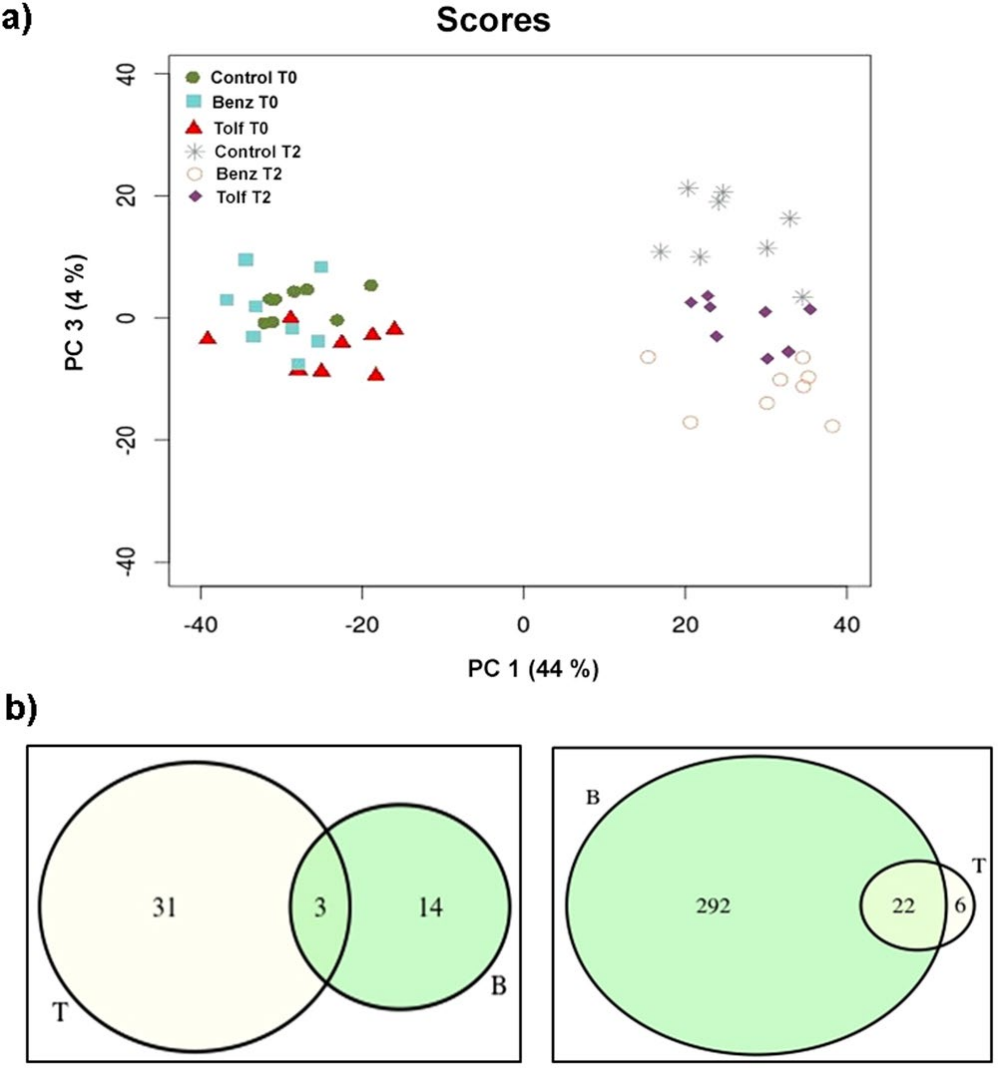
Differential gene expression upon Benz and Tolf treatments. (**a**) Principal component analysis (PCA) of the diversity of gene expression in treatment groups. PC 1 allows discrimination among seasons (T0 and T2). PC 3 allows discrimination between the three treatment conditions (Benz, Tolf and Control) as indicated. No significant variation was observed between the three treatment conditions. (**b**) Venn diagrams of downregulated (left) and upregulated (right) genes following treatment with Tolf (T; yellow circle) or Benz (B; green circle).

Principal Component Analysis (PCA) revealed that the greatest extent of variability in the data (PC1) was associated to the time of sampling and not to the treatment, which indicates that the major gene expression changes possibly reflect seasonal effects. Interestingly, PC3 discriminated treated samples and suggests a stronger effect of Benz than Tolf on gene expression (Fig. 6a).

We computed differential gene expression before and after treatments, controlling for seasonal differences accounted by the control trees. We obtained 34 down-regulated and 28 up-regulated genes for the Tolf treatment, while Benz induced the expression of 387 genes and down-regulated expression of 17 genes (Paired t-test with Fold Discovery Rate <0.05, (Fig. 6b). Notably most genes upregulated by Tolf were also induced by Benz, while little overlap on gene expression differences was observed for down-regulated genes, which suggests a consistent drug effect on the expression of citrus induced genes. To understand which cellular processes were affected by Benz and Tolf treatments, we performed pathway enrichment analysis over the set of differentially expressed genes using a Fisher’s Exact Test (FET). The pathway analysis, was done by applying the Fisher’s Exact Test (FET) implementation of the Paintomics and Kegga programs, and the Gene Set Enrichment Analysis (GSEA) implemented in the GSEA R package. In trees that received Benz, FET returned fifteen enriched pathways including phenylpropanoid biosynthesis, starch and sucrose metabolism, basic metabolic processes (lipids, nucleic acids and aminoacids), as well as pathways associated to biosynthesis/modification of the cell wall (Table 1). On the contrary, no enriched pathways were identified for the upregulated genes in trees that received Tolf, possibly due to the lower number of genes significantly induced/repressed in this second treatment.

**Table 1:** Enriched pathways using Fisher’s Exact Test of overexpressed genes in *Citrus clementina*, after treatment with Benzbromarone. Paintomics and Kegga analysis.

Pathway	Total Pathway Genes	Upregulated Genes	BH Adjusted p-value
Phenylpropanoid biosynthesis	135	10	3.22E-05
Phagosome	73	8	4.77E-05
Pentose and glucuronate interconversions	55	6	4.37E-04
Starch and sucrose metabolism	155	10	4.94E-04
Amino sugar and nucleotide sugar metabolism	110	8	8.20E-04
Cutin, suberine and wax biosynthesis	23	3	7.72E-03
Glycosaminoglycan degradation	12	2	1.87E-02
Fatty acid metabolism	57	4	1.90E-02
Fatty acid biosynthesis	37	3	2.82E-02
Cyanoamino acid metabolism	40	3	3.45E-02
Pentose and glucuronate interconversions	55	6	2.80E-06
Biosynthesis of secondary metabolites	928	19	2.49E-05
Phenylalanine metabolism	48	3	5.24E-03
Glycerophospholipid metabolism	73	3	1.65E-02
Carotenoid biosynthesis	34	2	2.58E-02

To investigate if Tolf affected the regulation of specific pathways at a lower magnitude, the transcriptomics results were examined using the more sensitive GSEA method. This method uses a ranking of genes associated to the treatment rather than a set of p-value selected genes. Seventeen enriched pathways were identified (Table 2). Interestingly, most of the pathways identified in trees that received Tolf coincided with those identified in Benz treated trees. These results indicate that both treatments triggered a similar transcriptional response, but of higher magnitude in Benz than in Tolf treated trees. No additional pathways were identified by GSEA of the Benz treatment data. From these analyses we concluded that both drugs triggered a mild and similar transcriptional response in the treated citrus trees, with a stronger effects for Benz. Trees were mostly affected at metabolic, secondary metabolites and cell-wall synthesis pathways.

**Table 2:** Gene set enrichment analysis (BH correction) of the transcriptional upregulated response of *Citrus clementina* treated with Tolf. The GSEA function in R was used.

Pathway	p-value
Carbon metabolism	2.28E-06
Ascorbate and aldarate metabolism	1.87E-04
Protein processing in endoplasmic reticulum	1.87E-04
Phenylpropanoid biosynthesis	1.93E-04
Photosynthesis	2.56E-04
Biosynthesis of amino acids	3.91E-04
Photosynthesis - antenna proteins	1.09E-03
Ribosome	1.09E-03
Phagosome	1.09E-03
Carbon fixation in photosynthetic organisms	2.19E-03
Glyoxylate and dicarboxylate metabolism	2.43E-03
Glycine, serine and threonine metabolism	3.52E-03
Glycolysis/Gluconeogenesis	3.55E-03
Amino sugar and nucleotide sugar metabolism	5.65E-03
Citrate cycle (TCA cycle)	7.18E-03
Cyanoamino acid metabolism	8.50E-03
Starch and sucrose metabolism	8.50E-03

### Evaluation of the efficacy of Benz and Tolf treatments on commercial groves

The results of the small-scale field trial indicated that each treatment consistently induced genes associated with normal metabolism and growth, without compromising tree viability or negatively affecting the indigenous citrus-associated microbiota. Additionally, no symptoms of phytotoxicity were observed in any of the treated trees. Given these results, additional field trials were conducted in actively producing commercial groves, to examine the effect of each treatment on fruit productivity, juice quality and efficacy against CLas.

**Figure 7:**
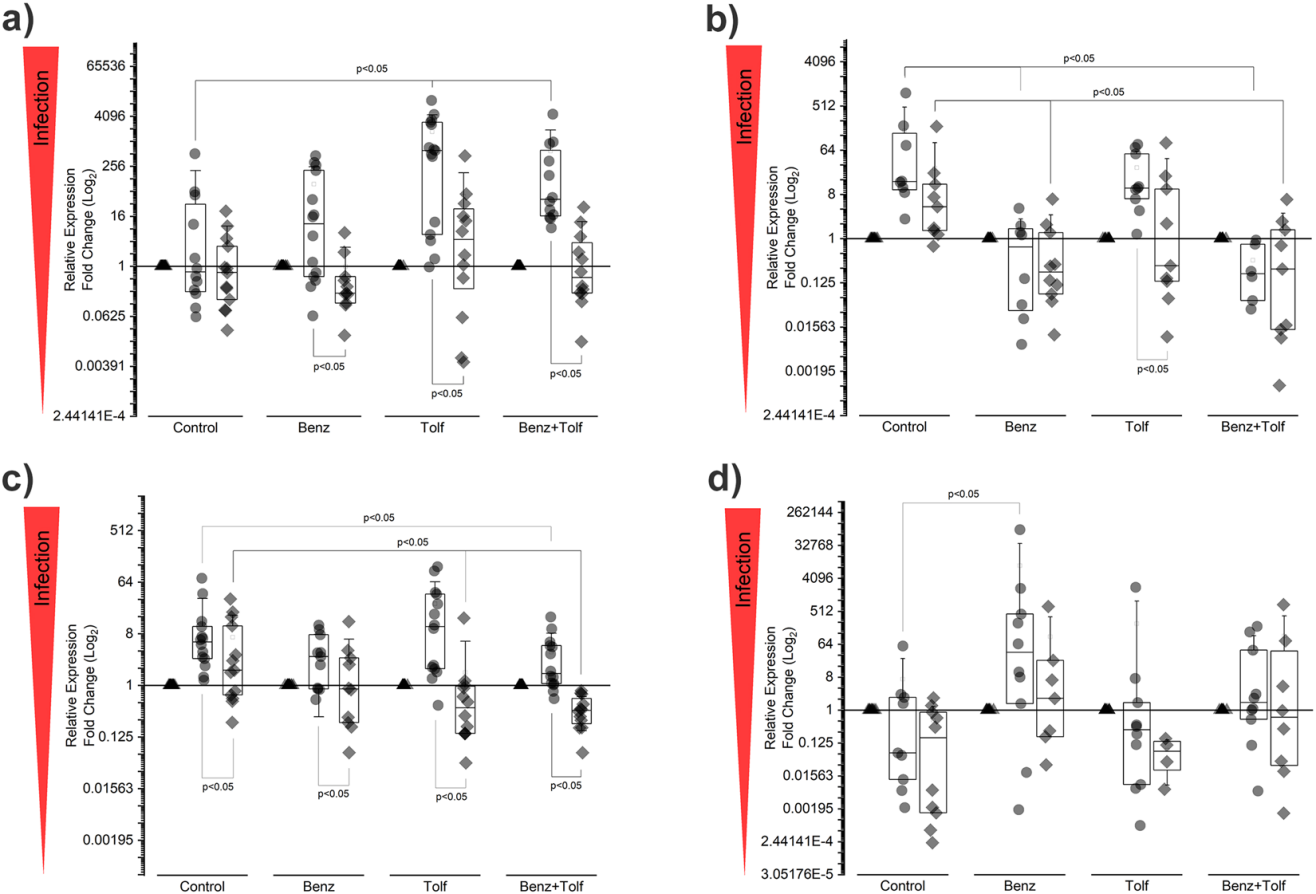
Evaluation of antimicrobial efficacy in commercial groves. Viability of CLas was evaluated in *C. sinensis* (**a**,**b**) and in *C. paradisi* (**c,d**) groves. The tissues analyzed were leaves (panels a and c), and roots (panels b and d) for time points T0 (▲), T6 (●) and T12 (◆) as indicated. ANOVA and Tukey’s post-hoc test was performed for each time point; only relevant statistical differences are shown.

Two field trials were conducted in Florida, where Benz, Tolf and the combination of Benz+Tolf were evaluated for efficacy against CLas in sweet orange (*C. sinensis*) and white grapefruit (*Citrus paradisi*) trees, ranging from 8 to 12 years old. Treatments were delivered by trunk injections (1,000 ml per tree) using a large-volume pneumatic injection system. Foliar and fibrous root tissue samples were collected prior to the first injection (March 2016, T0), and at three-month intervals thereafter (T3, T6 and T12, respectively). Seasonal variations in CLas transcriptional activity were evident in samples collected during field trials (Fig. 7). In *C. sinensis*, a significant decrease in CLas infection compared to the control (p < 0.05) was observed only in root tissue of trees that received Benz or Benz+Tolf injections (Fig. 7b), similar to the results observed in the mature trees (Fig. 2b). CLas viability measured by qRT-PCR in foliar tissue did not show a significant effect. However, a greater seasonal (T6 to T12) decrease in CLas activity was observed in all treatments (96-fold, 36-fold, and 146-fold for Benz, Tolf and Benz+Tolf, respectively) over time compare to the control (16-fold decrease) (Fig. 7a). Surprisingly, in *C. paradisi*, a significant decrease in CLas activity was observed in foliar tissue of trees that received Tolf and Benz+Tolf treatments when compared to the control (Fig. 7c). No significant effects were observed on root tissue (Fig. 7d).

### Evaluation of the effect of each treatment on fruit production and juice quality

Following twelve months of injection therapy, the fruit from each treatment group was quantified and analyzed for quality by measurement of mass, juice content, acidity, brix, total soluble solids, color and oil.

#### C. paradisi

The total yield, percentage of processable fruit (indicated with asterisk in Table 3), fruit drop, and fruit size frequency were recorded for each tree. The analysis of fruit yield was conducted using the collective total from each treatment group (n = 25 trees per group). Of the four treatment groups, trees that received Benz+Tolf injections produced the most processable fruit (3,214 pieces; 1,142 kg fresh weight) at the time of harvest. Trees that received Tolf injections were found to yield the second highest amount of processable fruit (3036 pieces; 1,093 kg fresh weight), while trees that received Benz or buffer (control) injections had the lowest fruit yields (919 and 985 kg fresh weight, respectively). Trees that received Benz+Tolf or Tolf injections had the lowest recorded fruit drop (7.5% and 7.6% drop, respectively), while a higher percentage of fruit drop was observed in trees that received Benz or buffer injections (8.6% and 8.9% fruit drop, respectively). This percentage was calculated from the total number of processable fruit collected from each treatment group at the time of harvest. There was no significant difference in fruit drop for individual trees between treatment groups (p > 0.05).

**Table 3:** Effect of each treatment on grapefruit size classification.

Grapefruit Size Classification	Minimum Fruit Dia. (inches)	Maximum Fruit Dia. (inches)	Control (%)	Benz (%)	Tolf (%)	Benz + Tolf (%)
>64	N/A	2 3/4″	25	25	17	19
64*	2 7/8″	3 1/8″	25	22	24	23
56–48*	3 1/4″	3 1/2″	26	23	27	28
40–36*	3 5/8″	3 7/8″	17	22	23	21
32–27*	4″	4 3/8″	5	6	7	7
<27*	4 1/2″	N/A	2	2	2	2

All fresh grapefruit were separated into groups according to their size (diameter) as indicated. The grapefruit size classification assigned to each group is based on the number of fruits per 4/5 – bushel container per the USDA NASS classification (2017). The fruit harvested in each category is expressed in percentage of the total hanging fruit for each treatment. (*) Denotes processable fruit categories.

The market value of grapefruit is determined by the fruit size. Consequently, the fruit size frequency was also recorded at the time of harvest for all *C. paradisi* trees. Fresh fruit was separated into groups according to size (diameter) as indicated in Table 3. The total weight and number of fruits within the cutoff for each size category was subsequently used to determine the fruit size frequency distributions for each treatment group.

It was found that trees injected with Benz, Tolf or Benz+Tolf had a higher frequency of size 40–36 fruit, when compared to the control group (Table 3). The highest percentage of unprocessable fruit (≤ 2^3^/_4_”) was observed with trees that received buffer control (25%) or Benz (25%) injections (Table 3). The percentage of fruit larger than four inches in diameter (size 32) was similar among all treatment groups.

Ten kilograms of fruit was collected from each tree for analysis at the Citrus Research and Education Center (CREC) Processing and Packinghouse Pilot Plant in Lake Alfred, Florida. The analysis included juice weight, juice per box, percent acid, brix (percent weight of soluble solids), pound solids (soluble solids in one box of fruit), and brix/acid ratio (Supplementary Table S2). No significant difference (p > 0.05) was observed between treatment groups for any of the parameters measured.

#### C. sinensis

The fruit from each treatment group (25 trees per group) was manually harvested, counted and weighed (Table 4). The statistical analysis indicated a large variability within and between treatment groups, thus no significant difference was observed between individual trees for the parameters analyzed. Noteworthy, the cumulative fruit yield of the Benz+Tolf treatment group was 3% higher than that of the control group (2,775 and 2,697 kg fresh weight, respectively). In addition, we recorded a decrease in fruit drop in treated trees, when compared to the controls. The juice yield and quality analysis conducted at the CREC indicated there were no significant differences for any of the parameters measured; juice color and oil analysis were also similar to the control group (Supplementary Table S3).

**Table 4:** Effect of the treatments on the *C. sinensis* fruit yield.

Treatment Group	Total Fruit Count	Fruit Count Per Tree	Total Yield (kg)	Yield Per Tree (kg)	Total Fruit Drop	Avg. Fruit Drop Per Tree*
Control	17503	700 ± 246	2,697	108 ± 38	343	13 ± 7
Benz	17650	706 ± 220	2,751	110 ± 34	278	11 ± 6
Tolf	17177	687 ± 232	2,639	105 ± 36	252	10 ± 6
Benz + Tolf	18393	735 ± 251	2,775	110 ± 40	295	11 ± 7

*Fruit drop counted at the time of harvest.

### LC-MS/MS analysis of benz and tolf in citrus tissue

LC-MS/MS was used to examine the concentration of Benz and Tolf in samples collected after 10 days or 30 days post injection on leaf tissue. The residual concentration of each compound was significantly lower after 10 days of injection in foliar tissue extracts (Benz = 57.5 ± 4 ng/g; Tolf = 46.5 ± 6 ng/g), while both compounds were below the limit of detection (LOD 4.0 ng/g) after 30 days. The chemicals were under the detection limit in root tissue (LOD = 3 ng/g) collected 30 days post injection. Similarly, the chemicals were below the detection limit in fruit, juice and soil tissue tested (LOD = 3 ng/g).

## Discussion

In this study, we present the results obtained when benzbromarone and/or tolfenamic acid were examined for efficacy against CLas viability in a greenhouse setting, as well as in a large field study in sweet orange (*C. sinensis*) and white grapefruit (*Citrus paradisi*) trees using trunk injections as a delivery mode. Two large-scale field trials were conducted in commercial citrus groves in Sebring (*C. sinensis*) and Fort Pierce (*C. paradisi*). In *C. sinensis*, a significant decrease in CLas activity was observed in root tissue of trees that received benzbromarone and the combination of benzbromarone and tolfenamic acid. Although no significant decrease was observed in *C. sinensis* foliar tissue samples, all treatments (benzbromarone, tolfenamic acid and the combined treatment) demonstrated a greater fold decrease in CLas viability than the controls. The fruit harvested from these trees also showed a noteworthy increase (3%) in cumulative fruit yield, and decreased (14%) fruit drop was observed in trees that received the combined (benzbromarone and tolfenamic acid) treatment, compared to the control group. In *C. paradisi*, a significant decrease in CLas activity was observed in the foliar tissue of trees that received tolfenamic acid or the combined benzbromarone and tolfenamic acid treatments. After twelve months of treatment, trees that received the combined treatment were also found to yield 16% more processable fruit than control trees.

The results obtained during field trials were modest when compared to the results from the greenhouse experiments that were conducted with graft-inoculated two-year-old citrus trees, albeit using different delivery methods. In our preliminary assays, it was determined that CLas titers were reduced in root and foliar tissue following treatments with tolfenamic acid^14^, and in foliar tissue with the combined benzbromarone and tolfenamic acid treatment (Fig. 1c). In the field trials reported here, only the roots showed a significant decrease in CLas titer, however, elimination of the bacteria was not observed. A possible explanation for these results is that upward movement/distribution (via translocation) of the injected chemicals was limited by the extent of HLB infection in the full-size, field grown trees, which were eight to ten years old when treatments began. It is well documented that the phloem cells of HLB-infected trees become obstructed by callose, P-protein plugs and starch grains, resulting in reduced and/or blocked flow within phloem conduits^29–33^. The possibility that phloem obstructions may have prevented the translocation of injected chemicals is also supported by the microbiome analysis, where the log2 fold change in *Liberibacter* abundance was suppressed in root tissue (p = 0.001) (Fig. 5).

Transcriptomic evaluation of the metabolic pathways in treated plants suggested the chemicals did not harm the trees or trigger pathways that would affect tree health or fruit quality. These results were confirmed by analysis of the fruit yield and juice quality, which showed no improvements or detrimental effects in either of the varieties tested (*C. sinensis* or *C. paradisi*). LC-MS analysis also confirmed that the injected chemicals were below the limits of detection in juice samples collected after 12 months of treatment.

Interestingly, the amount of *C. paradise* fruit classified in categories of higher marketable value significantly increased after the treatments; the combined benzbromarone and tolfenamic treatment was the most effective (Table 3). Considering that treatments were only administered for one season, we found these results to be remarkable, as reduced fruit size is one of the primary adverse effects of citrus greening disease. The fruit yield between individual trees was not found to meet statistical significance; however, differences in fruit production were evident among treatment groups. Trees that received injections formulated with the combined treatment (Benz + Tolf) were found to produce 15% more fruit (by fresh fruit weight), when compared to the control group. *C. paradisi* trees that received tolfenamic acid trunk injections had seven percent more fruit at the time of harvest, when compared to controls that received buffer only. These results are encouraging, as this trend could be significant when enlarging the sample size to eliminate variability from environmental factors. It was not possible to analyze the fruit harvest from the second season because the orchards were severely damaged by hurricane Irma in September of 2017.

The lower efficacy observed with benzbromarone and tolfenamic acid in field trials, when compared to greenhouse trials, may be due to the stability of each chemical under the conditions tested. The residual amount of each chemical was determined by LC-MS at 10 days and 30 days post-injection; results suggested that both chemicals were highly unstable in trees exposed to environmental factors. Further analysis suggest tolfenamic acid may be subject to inactivation/degradation upon exposure to light, with UV light potentially resulting in the formation of hydroxylated compounds of low toxicity, fostering further biological degradation^34^. In the literature, there are no scientific records regarding benzbromarone degradation by environmental factors; however, our results indicated a rate of degradation similar to that of tolfenamic acid, which may have been a major contributing factor in the reduced efficacy observed during field trials.

The reinfection rate of field-grown trees may have also contributed to the observed differences in efficacy during field trials. While previous greenhouse trials were performed in a psyllid-proof facility, field trials were conducted in open-air commercial citrus groves, where *Diaphorina citri* (Asian citrus psyllid) populations were near historical highs (Supplementary Fig. S2). Based on our field observations, we examined data collected by the Citrus Health Management Area (CHMA) regarding the psyllid populations in the areas where our field trials were conducted; the CHMA website includes reports with the number of *D. citri* collected in insect traps for each management area. The results indicated that the presence of *D. citri* in both field trial locations (CHMA 35S31E22-Lorida and 33S37E36-Indian River) duplicated during the last 4–5 months of the assay (Supplementary Fig. S2).

A number of publications described the use of different compounds against citrus greening disease, including classic antibiotics that are frequently used to treat bacterial infections in animals and humans^7,8,35^. With the exception of β-lactam antibiotics, no treatments have been fully effective in clearing CLas from infected trees^8^; however, β-lactams are not approved for use in citrus. Furthermore, the magnitude of antimicrobials that would be required to treat the vast amount of citrus that is currently in production could potentially generate a plethora of problems, including increased antimicrobial resistance and health associated problems (like allergic reactions) in rural communities. The US Environmental Protection Agency (EPA) is in the process of allow the use of two antibiotics on citrus, oxytetracycline and streptomycin^6,36^. These compounds will be applied using sprays systems and it is estimated that around 400,000 kilograms of each compound will be needed to treat the existing citrus groves in Florida alone^6,36,37^. More fascinating yet is that there is no published data with consistent evidence of the effectiveness of these treatments.

The *Liberibacter* genus has emerged as a group of endophyte bacteria capable of infecting a wide range of economically important crops^29^. The citrus greening epidemic affecting orchards worldwide is in urgent need of an effective treatment. The compounds analyzed demonstrated the ability to be efficient in a greenhouse setting with strong and specific interactions at a molecular level^13,14^. Improving the stability of these compounds through chemical engineering may help to increase the residual activity in field applications, however, strict protocols must be followed to effectively evaluate and compare the properties of newly derived compounds. The protocols used by our group could serve as a model to assess the efficacy of potential therapeutic compounds in field trials.

## Materials and Methods

### Evaluation of phytotoxicity

Formulations containing benzbromarone (Benz), tolfenamic acid (Tolf), or the combination of benzbromarone and tolfenamic acid (Benz + Tolf), were examined for phytotoxicity in two-year-old healthy ‘Valencia’ sweet orange trees (*Citrus sinensis* [L.] Osbeck), on Kuharski rootstock. Trunk injections were applied at increasing volumes (1–60 ml) and concentrations (0–25 mM). Benzbromarone (Benz) and tolfenamic acid (Tolf) were examined individually and in combination (Benz + Tolf). Trunk injections were administered using a custom-built pneumatic trunk injection system (Fig. 1a,b) that was designed in our laboratory specifically for this application (Gardner C., Gainesville, FL). All trees were observed for symptoms of phytotoxicity (thinning, color change, necrosis, deformation, etc.) for a period of twelve months after treatment. No phytotoxicity was observed at concentrations up to 2 mM, when the compounds were delivered individually or in combination.

### Evaluation of efficacy in greenhouse assay

The efficacy of each compound was examined in a greenhouse study at the University of Florida in Gainesville (Florida, USA) main campus, using graft-inoculated *C. sinensis* trees. Two-year-old citrus trees were graft-inoculated with HLB-infected budwood as previously described^14^. After testing positive for HLB infection for a period of six months, the graft-inoculated trees were split into groups of ten and treated with Benz (2.5–250 µM) and/or Tolf (2.5–250 µM), using a pneumatic trunk injection system (Fig. 1a,b). The control group consisted of ten trees that were injected with the buffer vehicle only. Following treatment application, root and foliar tissue samples were collected at three-month intervals for analysis by quantitative reverse transcription-polymerase chain reaction (qRT-PCR).

### Small-scale field site and experimental design

The small-scale field trial was conducted at the University of Florida main campus, in Gainesville (Florida, USA). Small molecules were delivered by trunk injections to twenty-four *C. sinensis* trees, ranging in age from twenty to thirty years old in advanced stage of HLB disease. Treatments were set up in a randomized complete block design, with three treatments in eight blocks (eight replications per treatment).

### Small-scale field trial trunk injections and tissue sampling interval

The goal of this experiment was to scale-up the chemical treatments from the small greenhouse trees, to large mature trees in the field, while minimizing phytotoxicity. To this end, four trunk injections were delivered over a twelve-month period with increasing concentrations of Benz or Tolf. The first treatment was delivered by trunk injection on April 1^st^, 2015 (day 0), where 1,000 ml of Benz (100 µM) or Tolf (100 µM) was delivered to each tree through three injection sites. The second treatment was delivered at day 64, where 3,000 ml of Benz (1 mM) or Tolf (1 mM) was delivered to each tree. The third treatment was delivered at day 175, where 3,000 ml of Benz (1.5 mM) or Tolf (2.5 mM) was delivered to each tree. The fourth treatment was delivered by trunk injection at day 385, where 1,000 ml of Benz (2.36 mM) or Tolf (3.82 mM) was delivered to each tree. Tissue samples (fibrous root and mature leaves) were collected from each tree the day before each injection treatment was delivered, and six months after the final injection.

### Large-scale field sites and experimental design

Two large-scale field trials were conducted independently in commercial groves located in Sebring and Fort Pierce, Florida (USA), from March 2016 through March 2017. Treatments in the Fort Pierce grove were applied to 100 HLB-infected white grapefruit trees (*Citrus paradisi*) grown on X639 rootstocks, ranging in age from eight to ten years old. Treatments in Sebring were applied to 100 HLB-infected *C. sinensis* trees grown on Swingle rootstocks, ranging in age from nine to twelve years old. Treatments in each grove were set up in a randomized complete block design, with four treatments in twenty-five blocks. Standard grove care and maintenance was performed in each grove throughout the field trials, which included irrigation, fertilization and standard pest management.

### Large-scale field trial trunk injections

A large-volume pneumatic trunk injection system was custom built in our lab (Gardner C., Gainesville, FL) to deliver compounds of interest to mature, HLB-infected citrus trees. The use of this system allowed us to injected large groups of trees (1–50) simultaneously. Trunk injections (1,000 ml per tree) were delivered at ninety-day intervals, over a period of twelve months. Injectants were delivered through four injection sites, spaced evenly around the base of each tree, at a distance of 150 to 200 mm above the soil line. Prior to drilling holes for injection ports, the base of each tree was sprayed with 10% bleach solution. All injection sites were sealed with pruning spray (post-injection). Chemical stocks were prepared in DMSO, and subsequently diluted with Tris buffer. All injection solutions contained 1% DMSO and 10 mM Tris (pH 8). Chemical treatment groups were injected with one liter of buffered solution containing benzbromarone (2.36 mM), tolfenamic acid (3.82 mM), or the combination of both compounds (2.36 mM Benz and 3.82 mM Tolf), as indicated. Control trees were injected with one liter of 10 mM Tris (pH 8.0), 1% DMSO. All trunk injections were conducted on clear days to promote the uptake of injectants by transpiration.

### Tissue sample collection for large-scale and small-scale field trials

Foliar and root tissue samples were collected using 50 mL PolyCarbonate Cryovials from Spex SamplePrep on the designated timepoint, from each tree in the experimental block. Due to the asymmetrical nature of HLB infection, the canopy of each tree was divided into four quadrants and ten mature leaves (whole) were randomly selected from each quadrant. Fibrous root samples were collected from three soil cores (5 cm diam., 25 cm depth), taken within the root zone of each tree (within one meter of the base of the trunk). The core samples were screened with 5 mm wire mesh to separate root tissue from the sand and soil substrate. The samples were immediately placed in a cooler, on dry ice, until processing.

### Tissue processing

Frozen tissue samples (foliar or fibrous root tissue) were lyophilized for 96 hours using a Labconco FreeZone 18 Liter Console Freeze Dry System. The samples were then ground at ~900 strokes/minute, using a 2000 Geno Grinder (Spex CertiPrep), with two sterilized 9.5 mm steel balls in each sample tube. Grinding was done in 30 seconds intervals, cooling the samples between intervals, until the sample became homogenized. Subsequently, a small amount of the homogenized tissue was transferred to a 5 mL polyethylene frosted screw cap tube with one sterilized 9.5 mm steel grinding ball, and ground at ~1200 strokes/minute for 45 seconds intervals until the samples were ground into a fine powder.

### RNA extraction and cDNA synthesis for qRT-PCR

25 mg of the powdered foliar tissue (from whole leaves) or fibrous root tissue was measured into a 2 ml screw cap tube for RNA extraction. RNA extraction was performed using the Zymo Research Direct-zol RNA MiniPrep kit, using the manufacturer’s recommended protocol. The RNA extract was then subjected to DNase treatment (Invitrogen DNase Turbo Enzyme). RNA samples were then quantified using NanoDrop ND-1000 Spectrophotometer and cDNA was synthesized using the BioRad iScript cDNA Synthesis kit for the leaf samples and NEB M-MuLV Reverse Transcriptase for root samples. All samples were normalized to 1 µg of total RNA and cDNA was synthesized following the protocol provided by the respective manufacturer for each kit.

### Real-time PCR

Quantitative reverse transcription-polymerase chain reaction (qRT-PCR) was performed with an Applied Biosystems Quantstudio 6 (ThermoFisher Scientific, Waltham, MA) using the synthesized cDNA template. TaqMan primers and probes for amplification of CLas 16S rRNA and citrus COX were previously designed and optimized^38^. The CLas 16S rRNA TaqMan probe contains FAM reporter and QSY7 quencher and the citrus COX Taqman probe contains VIC reporter and QSY7 quencher. The citrus gene encoding cytochrome *c* oxidase (COX) was used as a reference gene to normalize all qRT-PCR data. Each sample reaction was prepared in duplicates on a 384-well plate as described^12^ and analyzed on a QuantStudio 6 Flex, using comparative Ct method and standard protocols. Data was analyzed using the 2^−ΔΔCt^ method^39^.

### RNA-sequencing analysis on the citrus leaf

Three groups of eight infected trees (*C. sinensis* [L.] Osbeck) were established: control trees, trees treated with Benz and trees treated with Tolf. Leaf tissue samples were collected before the treatments began and after three months of treatment (one week after the second injection of Benz or Tolf).

Fifty leaves were collected randomly from the canopy of each tree. Following collection, the midrib and petiole of each leaf was excised and immediately submerged in RNAlater (Invitrogen, Carlsbad, CA). Prior to RNA extraction, the tissue samples were stored at −80 °C, and processed as previously described^13^. Total RNA was extracted with Isolate II RNA Plant Kit (Bioline, Taunton, MA). Purified RNA was stored at −80 °C prior to being shipped on dry ice for RNA-seq analysis at the Penn State College of Medicine Research Core Facility (Hershey, PA). RNA integration number (RIN) was measured using BioAnalyzer (Agilent Technologies) RNA 6000 Nano Kit. The cDNA libraries were prepared using the TruSeq Stranded Total RNA with Ribo-Zero Plant kit (Illumina) as per the manufacturer’s instructions. The unique barcode sequences were incorporated in the adaptors for multiplexed high-throughput sequencing. The final product was assessed for its size distribution and concentration using BioAnalyzer High Sensitivity DNA Kit (Agilent Technologies). Pooled libraries were denatured and loaded onto a TruSeq Rapid flow cell on an Illumina HiSeq. 2500 and run for 100 cycles using a paired-end read recipe according to the manufacturer’s instructions. Sequencing reads were de-multiplexed by Illumina CASAVA pipeline (version 1.8). In total, 48 samples were subjected to RNA-seq, and 2×101 bp and a total of 1,727,906,119 pair-end reads were obtained.

Quality of the sequencing reads were verified by FASTQC v.0.11.4^40^. Tophat v.2.1.1^28^ was used to align the reads to the *Citrus clementina* reference genome (ncbi_gca_000493195_1_citrus_clementina_v1_0), the *C. sinensis* chloroplast sequence, and the *Citrus clementina* mitochondrial genome. The choice of using the *Citrus clementina* as genome reference for read mapping instead of *C. sinensis* is because of the higher quality of the assembly of the *Citrus clementina* genome, compared to the *C. sinensis* genome. HTSEQ v.0.6.1p1^41^ was used for gene-level quantification of expression.

Genes with less than 10 reads collectively for all samples were discarded from further analysis. Gene expression values were normalized using the TMM method^42^ and structural artifacts, such as batch effects, were removed by ARSyNseq.^43^. Differential expression was computed using the R package limma v.3.3 and with data voom-transformed^44,45^. A paired test was applied using the tree as pairing factor and setting as contrast the difference in expression for treated trees at T0 (before treatment) and T2 (after treatment), relative to the differences for control trees at the same time points. Each treatment was evaluated independently, and significant genes were called at a FDR < 0.05. Gene Ontology (GO) terms for the genes in the *Citrus clementina* genome were annotated by Blast2GO v. 1.3.3^46^ and the Fisher’s exact test tool implemented in the software was used to test for functional enrichment within the sets of genes regulated by each chemical. Transcriptome data was further analyzed by the PaintOmics tool^47^ to locate the position of significant genes in pathways and select biomarkers for validation.

### Microbial community analysis

Root and leaf samples from treated *C. sinensis* [L.] Osbeck trees were collected as described previously^27^. Genomic DNA was extracted and Amplicon libraries were prepared for paired-end 2×150 cycles Illumina sequencing (MiSeq) as described in Blaustein *et al*.^27^. The reads were processed to define the OTUs and the taxas in each sample as described previously^27^.

Data analyses were performed in R v.3.2.1. Microbial community alpha diversity (Chao1 index and Shannon measure) was computed in phyloseq.^48^. Two-way ANOVAs were utilized to determine the effects of time and treatment on alpha diversity measures, the relative abundance of all *Alphaproteobacteria* and the relative abundances of genera within *Alphaproteobacteria*. Moreover, the associations of microbial community structure with treatment and with time were evaluated with Non-Metric Data Scaling (NMDS) plots and analysis of similarity (ANOSIM) (significance based on 999 permutations) in Vegan v.2.3.2^49^. Note that an ANOSIM R statistic of 0 means the communities are identical; whereas, R of 1 means the communities have no overlap. Changes in the relative abundance of *Liberibacter*, as well as other taxa, were calculated as log2 ratios for all time points relative to time 0 (i.e., log2 fold change). Since log transformation of zero values is undefined, samples with a 0% relative abundance were tallied as present with a relative abundance equivalent to 50% that of a singleton in the sample, as described in Amend *et al*.^50^. The time series for alpha diversity and that of the log2 fold changes in *Liberibacter* relative abundance were plotted with ggplot2^51^. A two-way ANOVA was utilized to determine the effects of time and treatment on the log2 fold changes in *Liberibacter*. Associations between log2 fold changes in relative abundances of the pathogen and those of bacterial families that had average relative abundances greater than 0.1% throughout the study were determined with regression analyses.

### Fruit harvest and analysis

Following twelve months of treatments in the large-scale field trial, the fruit from each tree was harvested and classified independently to determine the overall yield and size distribution for all hanging fruit, on each tree. Grapefruit were separated according to size (diameter) and subsequently grouped together as indicated in Table 3. Fruit droppage was recorded at three-month intervals throughout the study, to analyze the effect of each treatment on fruit retention.

Fruit samples (9.07 kg per tree) were submitted to the Citrus Research and Education Center (CREC) Processing and Packinghouse Pilot Plant, in Lake Alfred Florida (USA), for quality analysis. Samples were analyzed by the CREC according to the system developed by the Florida Department of Agriculture and Consumer Services (FADCS - USA). Oil analysis (Scott method^52^) and juice color was also performed for *C. sinensis* samples.

### Evaluation of compounds in plant tissue

LC-MS/MS was used to examine the concentration of benzbromarone and tolfenamic acid on leaves, roots, fruit peel, fruit juice and soil samples of *C. sinensis* [L.] Osbeck trees. Benz and Tolf were purified by organic extraction from freeze-dried tissue, and subsequently analyzed by HPLC and LC-MS/MS (Zhang *et al*., submitted for publication).

Briefly, lyophilized material was incubated for 1 hour at 25 °C with formic acid (0.1%) and ethyl acetate and n-hexane (6:4, v/v) were added to the mixture, followed by vortexing. Samples were then centrifuged at 17,000 g for 15 min, at 4 °C and the supernatant was transferred to a new tube and dried completely under nitrogen at 40 °C. The dried extract was reconstituted with ultra-pure water and acetonitrile (1 mL, 1:1, v/v). A volume of 5 µL was used for LC–MS/MS analysis. Gradient separation was done using a Waters Symmetry C18 column (150 mm ×2.1 mm, 3.5 µm). The mobile phase consisted of formic acid (0.1%) in water and formic acid (0.1%) in acetonitrile. The compounds were identified using TSQ Quantum Access MAX Triple Quadrupole system coupled with ACCELA 1250 UHPLC system. Analyses were carried out using heated-electrospray ionization in negative mode with selected reaction monitoring (Zhang *et al*., submitted for publication).

### Statistical analysis

A repeated measures analysis was performed on the response variable ΔΔCt of the qRT-PCR data from infected citrus leaves and roots. The linear mixed model considered a random effect of block, and fixed effects of time, treatment (Control, Benz, Tolf and Benz+Tolf) and their interaction. The repeated nature of the data was modeled using an autoregressive order 1 error structure, and Kenward-Rogers correction of the degrees of freedom were used for the analyses of variance. Multiple comparisons were obtained using least significance differences, and specific contrast were evaluated that compared the treatment level means between years and between specific months. All tests were done considering a significance level of 5%, and the statistical software used corresponded to SAS v. 9.4^53^.

In the microbial community analysis, two-way ANOVAs were utilized to determine the effects of time and treatment on alpha diversity measures and to determine the effects of time and treatment on the log2 fold changes in *Liberibacter* as it was described in the previous section.

In the juice yield and quality analysis, and in the fruit drop for individual tress, two-way ANOVAs were utilized to determine the effects of treatment.

## Supplementary information

Supplementary Information.

## Data Availability

The RNAseq datasets generated during and/or analyzed during the current study are available in the GEO repository, accession number GSE137736, https://www.ncbi.nlm.nih.gov/geo/query/acc.cgi?acc=GSE137736. The Microbiota community datasets generated during and/or analyzed during the current study are available from the corresponding author upon reasonable request.
