# Supplementary material for: Assessment of unconventional antimicrobial compounds for the control of ‘Candidatus Liberibacter asiaticus’, the causative agent of citrus greening disease
Source: Sci Rep. 2020 Mar 25;10:5395. doi: 10.1038/s41598-020-62246-x (PMC7096471; doi:10.1038/s41598-020-62246-x)
Supplement: Supplementary file 1 — Supplementary Information. [file 41598_2020_62246_MOESM1_ESM.pdf]

SUPPLEMENTARY INFORMATION

Assessment of unconventional antimicrobial compounds for the control of '*Candidatus* Liberibacter asiaticus', the causative agent of citrus greening disease

[1]*Christopher L. Gardner, [1]*Danilo R. da Silva, [1]Fernando A. Pagliai, [1]Lei Pan, [1]Kaylie A. Padgett-Pagliai, [2]Ryan A. Blaustein, [1]Marcelo L. Merli, [1]Dan Zhang, [1]Cécile Pereira, [2]Max Teplitski, [3]Jose X. Chaparro, [4]Svetlana Y. Folimonova, [1]Ana Conesa, [5]Salvador Gezan, [1]Graciela L. Lorca, [1]Claudio F. Gonzalez.

[1]Microbiology and Cell Science Department, Genetics Institute, Institute of Food and Agricultural Science, University of Florida, Gainesville, Florida, United States of America

[2]Soil and Water Sciences Department, Genetics Institute, Institute of Food and Agricultural Science, University of Florida, Gainesville, Florida, United States of America

[3]Fruit Tree Breeding and Genetics, Horticultural Sciences Department, Institute of Food and Agricultural Science, University of Florida, Gainesville, Florida, United States of America

[4]Plant Pathology Department, Institute of Food and Agricultural Science, University of Florida, Gainesville, FL 32611, USA.

[5]School of Forest Resources and Conservation, Institute of Food and Agricultural Science, University of Florida, Gainesville, Florida, United States of America

** These authors contribute equally to the present work.*

University of Florida, 2033 Mowry Road Lab 325, Gainesville, Florida, 32611

Phone: 352-273-8088, Fax: 352-273-8284, E-mail: cfgonzalez@ufl.edu

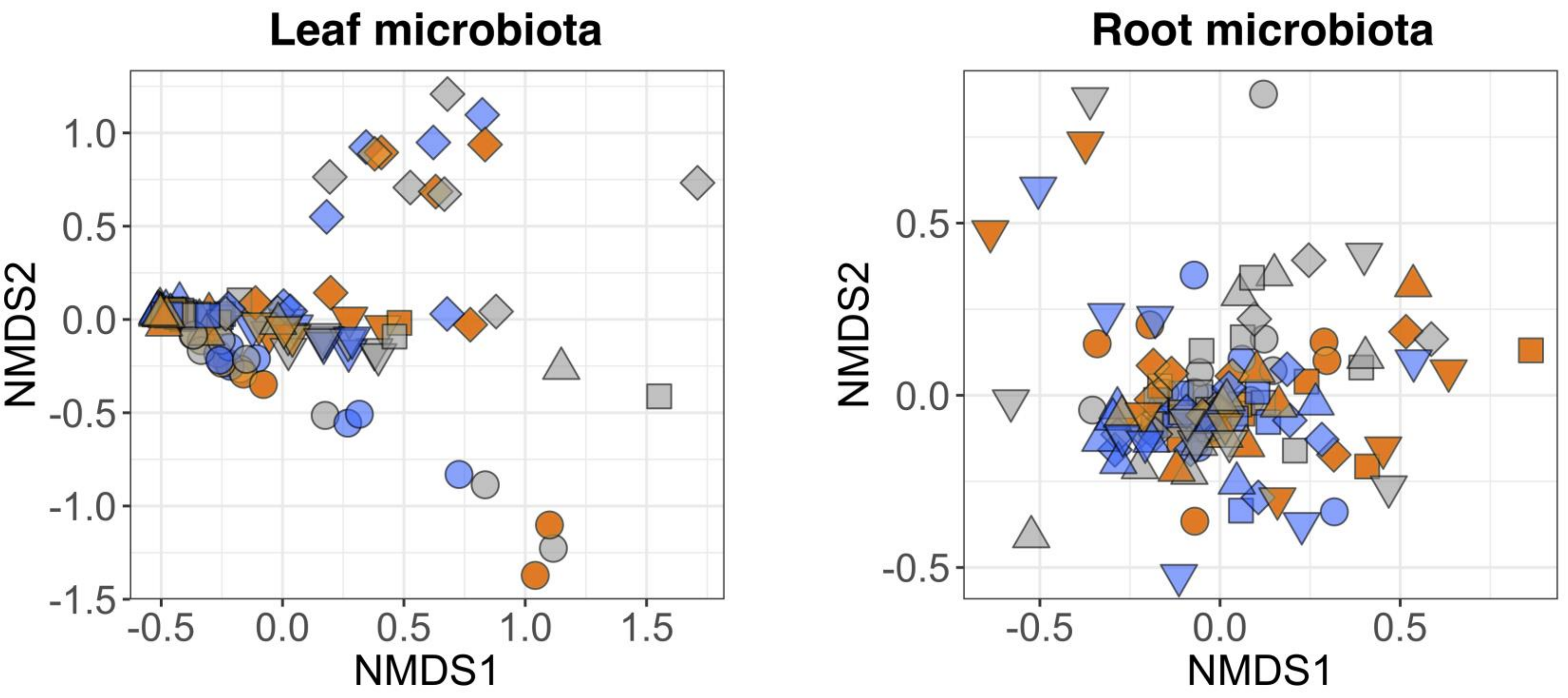

Supplementary Figure S1: NMDS plots for differences in the structure of leaf and root microbiota as a factor of sampling time before treatment (T0, ●) and 2, 6, 12 and 18 months post-treatment (T2, ■; T6, ♦; T12, ▲; and T18, ▼, respectively). Treatment groups are indicated by color as follows: Control (gray), Benz (blue), Tolf (orange).

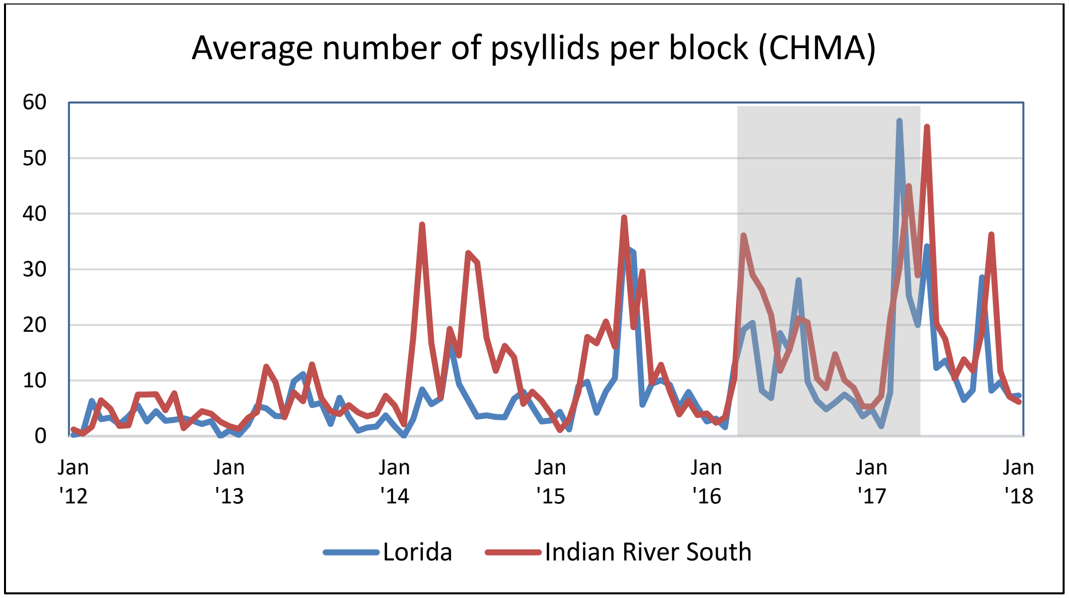

Supplementary Figure S2. Abundance of *Diaphorina citri* in field trial locations. The average Asian citrus psyllid (ACP) population in Lorida (blue) and Indian River South (red) from January 2012 through January 2018. Field trails were conducted in both locations, from March 2016 through March 2017 (period highlighted in gray). Average psyllid population data was obtained from the citrus health management area (CHMA) website (www.flchma.org).

Sample type	Treatment	Genus	Relative abundance in microbiota of control trees[a]	Relative abundance in microbiota of treated trees[a]	p(treat.)[b]	p(time)[b]	p(inter.)[b]
Leaf	Benz	Uncl. in *Sphingomonadaceae*	0.467 ± 0.089 %	0.301 ± 0.069 %	**0.023**	**<0.001**	0.119
	Tolf	Uncl. in *Sphingomonadaceae*	0.467 ± 0.089 %	0.257 ± 0.054 %	**0.019**	**<0.001**	0.160
		Uncl. in *Aurantimonadaceae*	0.226 ± 0.053 %	0.100 ± 0.024 %	**0.027**	**<0.001**	0.089
Root	Benz	*Agrobacterium*	2.535 ± 0.355 %	1.607 ± 0.197 %	**0.017**	0.332	0.584
		Uncl. in *Alphaproteobacteria*	0.942 ± 0.046 %	1.056 ± 0.043 %	**0.013**	0.653	0.736
		Sphingomonas	0.595 ± 0.074 %	0.404 ± 0.039 %	**0.040**	0.659	0.574
	Tolf	*Aminobacter*	0.0089 ± 0.0015 %	0.0044 ± 0.0011 %	**0.009**	0.388	0.257
		Rubellimicrobium	0.0026 ± 0.0009 %	0.0003 ± 0.0002 %	**0.044**	0.676	0.290

Supplementary Table S1. Genera within *Alphaproteobacteria* that significantly differed (p<0.05) in relative abundance based on treatment received, and corresponding p-values from a two-ANOVA evaluating differences based on treatment (Control vs. Benz or Tolf) time (0, 2, 6 12, 18 months), and interactions of treatment per time. For each genus listed, there were no differences for the three treatment groups at the start of the study (i.e., p>0.05 in two-tailed Student's t-test).

[a] mean ± SE for relative abundance at all times beyond initial treatment (2, 6, 12, and 18 months)

[b] analysis had been performed on the subset of genera within *Alphaproteobacteria* that had significant differences (p<0.05) based on treatment in a two-way ANOVA for differences based on treatment and time (i.e., the values may have differed between Benz and Tolf or between either compound and the Control).

Treatment	Juice per box (kg)	Acid	Brix	Brix/Acid ratio	Solids per box (kg)
Control	21.2 ± 1.2	1.18 ± 0.11	9.08 ± 0.58	7.77	1.93 ± 0.20
Benz	21.5 ± 1.0	1.17 ± 0.08	9.24 ± 0.47	7.94	1.99 ± 0.16
Tolf	21.6 ± 0.8	1.16 ± 0.08	9.19 ± 0.29	7.95	1.99 ± 0.11
Benz+Tolf	21.6 ± 1.0	1.18 ± 0.08	9.22 ± 0.62	7.86	1.99 ± 0.19

Supplementary Table S2: *Citrus paradisi* juice yield and quality analysis

Treatment	Juice per box (kg)	Acid	Brix	Brix/Acid ratio	Solids per box (kg)	Color	% Oil
Control	24.0 ± 0.8	0.9 ± 0.3	13.4 ± 0.6	15.1	3.22 ± 0.1	38.2 ± 0.92	0.23 ± 0.09
Benz	23.0 ± 1.0	0.9 ± 0.1	13.4 ± 0.6	14.8	3.20 ± 0.1	38 ± 0.89	0.20 ± 0.08
Tolf	23.0 ± 0.7	0.9 ± 0.4	13.5 ± 0.7	14.8	3.23 ± 0.2	38 ± 0.67	0.23 ± 0.07
Benz+Tolf	23.1 ± 0.7	0.9 ± 0.2	13.5 ± 0.5	15.0	3.24 ± 0.1	38.2 ± 0.82	0.22 ± 0.10

Supplementary Table S3: *Citrus sinensis* juice yield and quality analysis
